# Secondary glaucoma with anterior chamber cholesterolosis: Case series

**DOI:** 10.1097/MD.0000000000028655

**Published:** 2022-01-21

**Authors:** Ruijuan Han, Aijun Tian, Yaxin Fan, Juan Yin, Mingyu Ren

**Affiliations:** Department of Glaucoma, Hebei Eye Hospital, Hebei Provincial Key Laboratory of Ophthalmology, Hebei Provincial Clinical Research Center for Eye Diseases, Xingtai, Hebei Province, China.

**Keywords:** anterior chamber, case report, cholesterolosis, secondary glaucoma

## Abstract

**Rationale:**

The presence of cholesterol crystals in the anterior chamber is extremely rare, and secondary glaucoma with cholesterol crystals in the anterior chamber, reported in the literature, is even rarer. This paper reports 3 cases of secondary glaucoma with cholesterol crystals in the anterior chamber.

**Patient concerns:**

Three patients were admitted to the hospital because of ocular distension and blindness. Ocular examination on admission indicated high intraocular pressure, and crystalline gold substances were observed in the anterior chamber.

**Diagnosis:**

Based on clinical manifestations and an aqueous fluid smear, absolute glaucoma and anterior chamber cholesterol crystals were diagnosed.

**Interventions:**

In the first case, transscleral ciliary photocoagulation was performed; in the last 2 cases, trabeculectomy combined with extracapsular cataract extraction was performed.

**Outcomes:**

The follow-up period was 11 to 15 months. Intraocular pressure was stable in 2 patients treated with surgery, and no cholesterol crystals were observed in the anterior chamber. The intraocular pressure increased in 1 patient treated with laser, and a small amount of cholesterol crystals was still observed in the anterior chamber.

**Lessons:**

Anterior chamber cholesterol crystallization is extremely rare and cannot be treated if it does not cause other lesions. However, glaucoma occurred in all 3 cases in this study, and intraocular pressure increased in 1 case after laser treatment and remained stable in 2 cases after surgical treatment. Therefore, the treatment plan for anterior chamber cholesterol crystallization in glaucoma requires further discussion.

## 1 Introduction

Cholesterolosis bulbi is a degenerative end-stage sequela of certain ocular lesions, manifested by cholesterol crystallization mainly in the vitreous cavity, subretinal space, and occasionally in the anterior chamber.^[1]^ Cholesterol crystals may be derived from ocular blood breakdown products,^[2]^ commonly secondary to Coats’ disease, Eales disease, retinal detachment, retinitis pigmentosa, trauma, cataracts, chronic uveitis, bleeding, anterior chamber hematocele, and retinoblastoma.^[3–5]^ We herein describe 3 cases of secondary glaucoma with anterior chamber cholesterolosis.

## 2 Case report

### 2.1 Case 1

**Figure 1:**
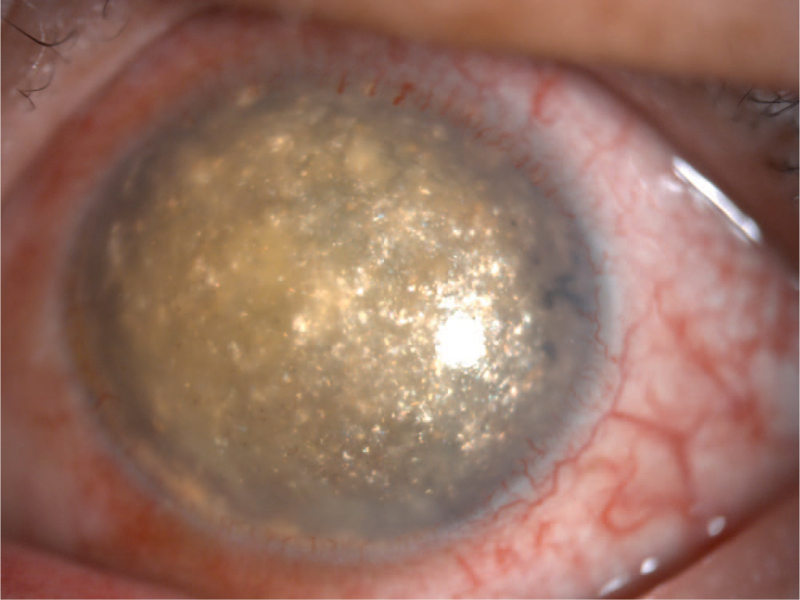
Gold crystal-like substances were appeared in the anterior chamber, in a 49-year-old east Asian male patient.

**Figure 2:**
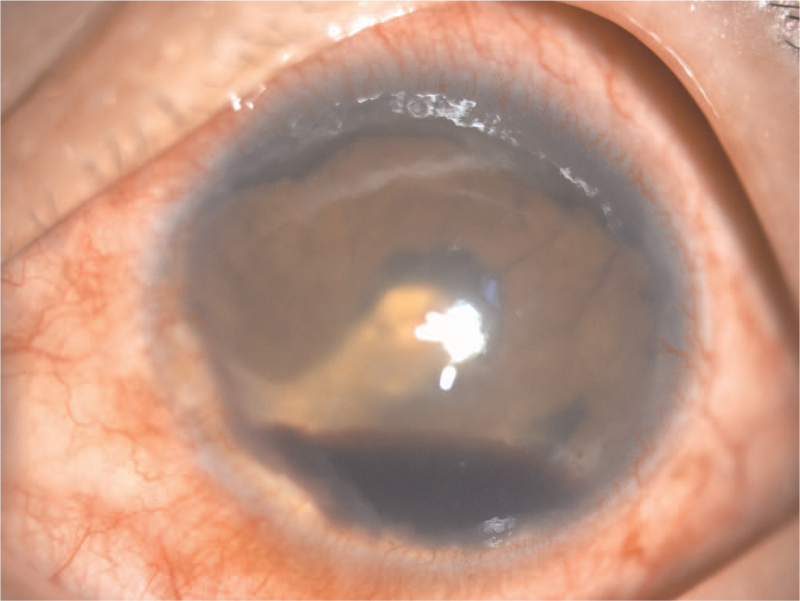
Flocculent material and blood was appeared in the anterior chamber, with lens cortex deposition on the iris surface, after upper iris root devascularization.

**Figure 3:**
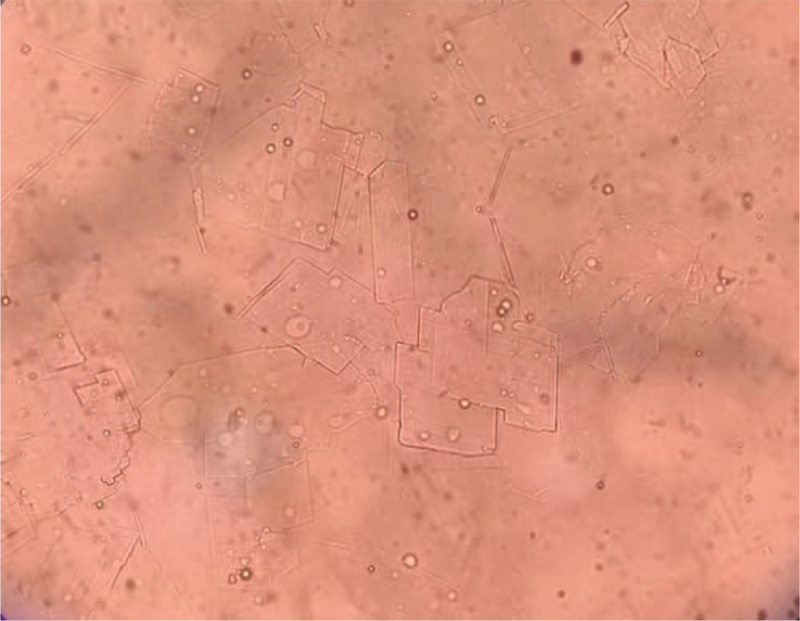
Cholesterol crystals were found on pathological examination (×40).

A 49-year-old East Asian man was admitted to our hospital with a 6-month history of redness, milling, and blindness in his left eye. The systemic evaluation was informative at the time of admission. The patient had a 30-year history of binocular myopia and denied ocular trauma or surgery. His right eye had a vision of 0.04, while his left eye had no light perception. The intraocular pressure (IOP) was 10 mm Hg in the right eye and >60 mm Hg in the left eye. The left eye had moderate conjunctival congestion, misty corneal opacity with abundant gold crystal-like substances attached to the posterior wall, a faint disappearance of the peripheral anterior chamber, and an invisible remaining eye structure (Fig. 1). An A/B-scan revealed high opacity of the left vitreous cavity, retinal detachment (cyst), and an acoustic image of bulbar edema with abnormal echogenicity (calcification). Ultrasound biomicroscopy revealed that the anterior chamber depth was 2.32 mm in the right eye and 1.32 mm in the left eye and abnormal morphology of the chamber angle in both eyes. Meanwhile, the left eye had aqueous humor opacity and unhealthy iris morphology. Orbital computed tomography revealed decreased lens density, narrowed anterior chamber space, increased vitreous density, and choroidal osteoma on the posterior wall of the eyeball in the left eye. Blood lipid tests revealed no abnormalities. The patient was diagnosed with secondary glaucoma of the left eye. On admission, the patient was assigned to receive IOP-lowering and anti-inflammatory medication. To better understand the intraocular conditions, needle aspiration was performed on the left anterior chamber, and the acquired aqueous humor was processed into smears for microscopic examination. Following needle aspiration, there was small blood loss from the anterior chamber, as well as upper iris root devascularization and visible flocculent and lens cortex deposition on the pupils and iris surface (Fig. 2). Smear microscopic examination revealed a large number of cholesterol crystals that were transparent, colorless, and shaped in rectangles or squares with notched corners (Fig. 3). Photocoagulation of the sclera and ciliary body was scheduled under local anesthesia. The IOP was well controlled after the operation, and only a few cholesterol crystals remained in the anterior chamber. The follow-up period was 15 months, and the patient's IOP in the left eye increased 6 months after discharge, but was medically controlled and stabilized after numerous reexaminations.

### 2.2 Case 2

**Figure 4:**
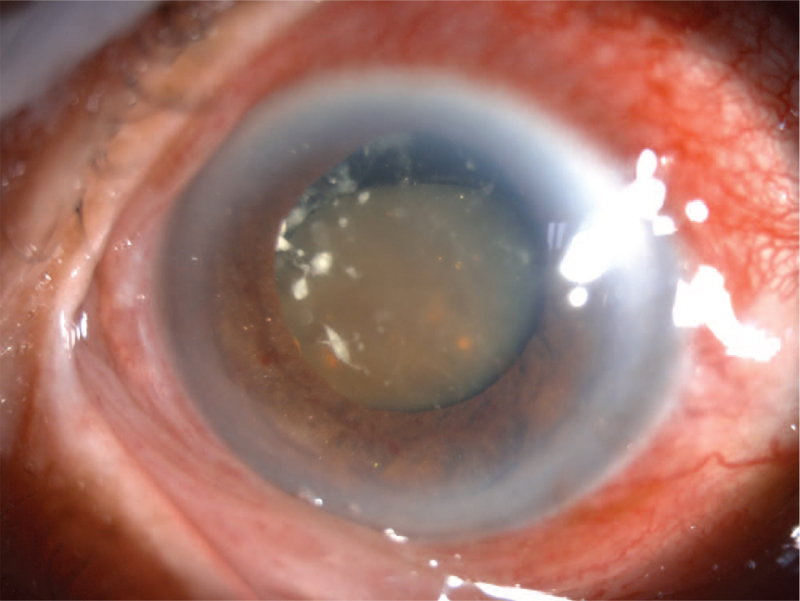
Gold crystal-like substances were appeared in the anterior chamber, in an 85-year-old Asian female patient.

An 85-year-old Asian female patient was admitted to our hospital with a 17-year history of poor visual acuity in the right eye and a 15-day history of swollen eye pain with ipsilateral headache. The systemic evaluation was informative at the time of admission. The patient had no light perception in the right eye, but the left eye had a vision of 0.4. The IOP was 15 mm Hg in the right eye and 12 mm Hg in the left eye. The right eye had moderate conjunctival congestion, light corneal edema, and normal anterior chamber depth (around 1/3 corneal thickness of the peripheral chamber angle) with some visible gold crystal-like substances floating, a round pupil measuring 6 mm in diameter and unresponsive to light, with the anterior lens capsule dispersed in white calcified flakes, cortex releasing, and brown nuclei deposited below and in a sun-setting shape (Fig. 4). An A/B-scan indicated vitreous opacity in both eyes (vitreous membrane of the right eye). Blood lipid tests revealed no abnormalities. The patient was diagnosed with secondary glaucoma of the right eye. The anterior chamber of the right eye was aspirated with a needle, and aqueous humor samples were smeared and examined microscopically. A small number of cholesterol crystals were transparent, colorless, and shaped as rectangles or squares with notched corners. On admission, the patient received IOP-lowering and anti-inflammatory treatments and subsequently underwent trabeculectomy for the right eye plus cataract extraction under local anesthesia to achieve a well-controlled postoperative IOP and clear aqueous humor in the anterior chamber. After a 12-month follow-up, IOP remained steady, and there was no sign of ocular discomfort.

### 2.3 Case 3

**Figure 5:**
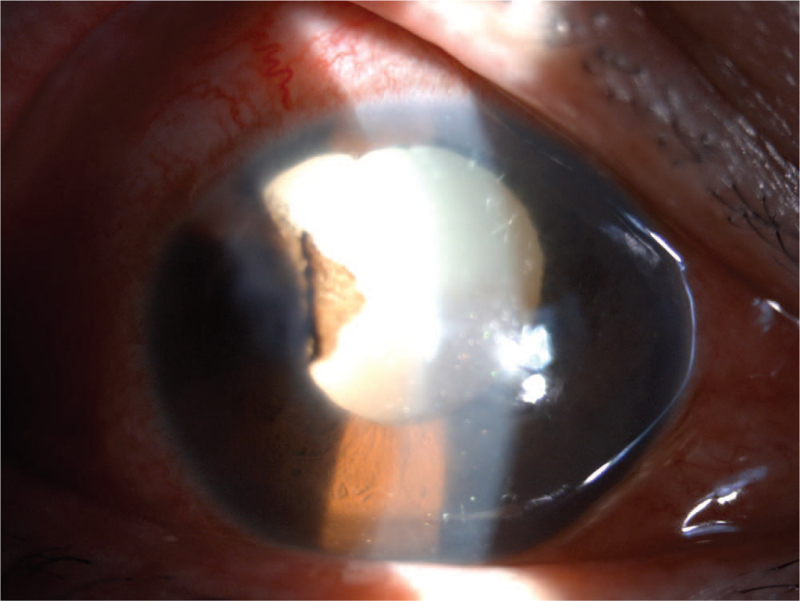
Gold crystal-like substances were appeared in the anterior chamber, in a 55-year-old Asian male patient.

A 55-year-old Asian man was admitted to our hospital with a 1-day history of redness, milling, and swelling pain in the right eye. The systemic evaluation was informative at the time of admission. The patient's right eye was injured by a bottle cap 50 years ago, and her visual acuity gradually deteriorated. The right eye had no light perception, whereas the left eye had a vision of 0.5. IOP was >60mm Hg in the right eye and 18 mmHg in the left eye. The right eye had moderate conjunctival congestion, visible grey-white flakes in the deep stromal layer at the temporal side of the central cornea, and anterior iris adhesion in the corresponding area, moderate edema of the remaining ocular region, normal anterior chamber depth while partially visible gold crystal-like substances floated, a subrounded pupil at 5 mm in diameter, which was brown, unresponsive to light, with chylous lens cortex, and deposited nucleus (Fig. 5). An A/B scan revealed vitreous opacity and posterior wall detachment in the right eye. Ultrasound biomicroscopy revealed an anterior chamber depth of 2.92 mm in the right eye and 2.63 mm in the left eye. Meanwhile, the right eye had aqueous humor opacity, anterior iris adhesion, lens opacity, and insufficient detachment. Blood lipid tests revealed no abnormalities. The patient was diagnosed with secondary glaucoma and age-related cataracts (after-ripened) in the right eye. On admission, the patient was assigned to receive IOP-lowering and anti-inflammatory medication. Intraoperative needle aspiration was performed in the right anterior chamber and the acquired aqueous humor was processed into smears. Microscopically, a small number of cholesterol crystals were observed. They were transparent, colorless, and shaped into rectangles or squares with notched corners. Trabeculectomy combined with cataract extraction in the right eye was performed under local anesthesia. Following the operation, the patient had well-controlled IOP and clear aqueous humor in the anterior chamber. After a 12-month follow-up, the IOP remained steady, and there was no sign of ocular discomfort.

## 3 Discussion

Cholesterol crystals usually form within the vitreous cavity, but cases in which cholesterol particles are formed in the anterior chamber have occasionally been reported.^[6]^ Cholesterol crystal deposition in the anterior chamber is very rare in the clinic, particularly in both eyes.^[1–5,7]^ In 1828, Parfait-Landrau first reported the formation of sparkling crystals in a human ocular vitreous body. Three years later, Schmidt discovered cholesterol crystals.^[3]^ Subsequently, numerous similar cases were reported. In anterior chamber cholesterolosis, both domestic and international reports indicated a relationship with blindness, but not with patient age, sex, place of residence, or biochemical indices such as blood cholesterol content.^[1–5,7]^

It is known that there are few cholesterol molecules in ocular tissues. Cholesterol crystals are a general degenerative outcome of trauma or inflammation and are largely present owing to the breakdown of old bleeding or long-standing exudates.^[8]^ Nonetheless, vitreous bleeding absorption insufficiency, lipidation, and chronic uveitis are also possible causes of cholesterol crystallization.^[7]^ If there is lens opacity in the clinical setting, the cholesterol content can increase tenfold, implying that phacolysis is also a contributing factor.^[9]^ The specific origin of the cholesterol crystals detected during phacolysis remains unknown. These may be byproducts of the lens membrane degradation. In previous studies on cases of after-ripened cataracts, cholesterol crystallization was also accompanied by iris neovascularization. In this population, some people believe that the formed cholesterol crystals were possibly caused by exudates or lost blood from new vessels.^[8]^ Additionally, in situ formation of cholesterol crystals in the anterior chamber has been observed to occur secondary to bleeding.^[10]^ Anterior chamber cholesterolosis occurs due to the breakdown of the anterior chamber hematocele and metabolites following the delivery of cholesterol crystals rich in subretinal hydrops and vitreous body to the posterior chamber via the degenerative suspensory ligament space.^[11]^ In the present study, cholesterol crystallization in case 1 was attributed to long-standing retinal detachment, and the resulting cholesterol crystals traveled to the anterior chamber via the degenerative suspensory ligament space. However, the cause of retinal detachment requires further investigation. In cases 2 and 3, cholesterol crystallization was considered a byproduct of phacolysis in the after-ripened cataracts.

Typically, anterior chamber cholesterolosis is diagnosed using a combination of clinical examination and crystal identification. Slit-lamp examination is a noninvasive, clinically significant diagnostic tool. Microscopic findings of anterior chamber cholesterolosis include transparent cholesterol crystals, which are shaped in rectangles or rhombi with characteristic concave corners and demonstrate birefringence (double refraction of polarized light).^[8]^

Secondary glaucoma is a common complication of anterior chamber cholesterolosis and has been previously reported.^[2,3,12,13]^ Cholesterol crystals are larger than calcium oxalate crystals, proteinaceous crystals, and aqueous cells, whereas proteinaceous crystals and aqueous cells are more susceptible to lysis.^[4]^ In this scenario, large cholesterol crystals located in the anterior chamber can pathologically increase IOP by blocking the outflow of aqueous humor. As a result, the 3 patients in the study were assumed to have glaucoma due to chamber angle obstruction induced by an overabundance of cholesterol crystals in the anterior chamber.

Currently, treatment options for anterior chamber cholesterolosis are limited. Moreover, as it might be associated with underlying disease progression,^[10]^ there is frequently no need for treatment.^[13]^ However, symptomatic treatment should be scheduled if pain or inflammation occurs, whereas additional treatment may be required when phacolysis occurs or glaucoma develops.^[7]^ All 3 cases developed secondary glaucoma. Case 1 underwent photocoagulation through the sclera and ciliary body without surgery due to iris root devascularization, retinal detachment, and unclear lens condition. He had IOP re-elevation 6 months after the operation because of the incomplete clearance of cholesterol crystals, which was considered to be caused by crystal-blocking chamber corners. The remaining 2 cases underwent glaucoma filtration surgery in conjunction with cataract extraction and had a well-controlled postoperative IOP due to complete removal of cholesterol crystals from the anterior chamber.

## Author contributions

**Conceptualization:** Ruijuan Han, Aijun Tian, Yaxin Fan.

**Data curation:** Juan Yin, Mingyu Ren.

**Formal analysis:** Yaxin Fan, Juan Yin, Mingyu Ren.

**Investigation:** Aijun Tian, Mingyu Ren.

**Methodology:** Ruijuan Han, Aijun Tian, Yaxin Fan.

**Project administration:** Ruijuan Han.

**Supervision:** Ruijuan Han, Aijun Tian, Mingyu Ren.

**Writing – original draft:** Ruijuan Han, Aijun Tian, Yaxin Fan.
